# Insights into the Pathogenesis of HS and Therapeutical Approaches

**DOI:** 10.3390/biomedicines9091168

**Published:** 2021-09-06

**Authors:** Elia Rosi, Maria Thais Fastame, Ilaria Scandagli, Antonella Di Cesare, Federica Ricceri, Nicola Pimpinelli, Francesca Prignano

**Affiliations:** Department of Health Sciences, Section of Dermatology, University of Florence, 50125 Florence, Italy; elia.rosi@unifi.it (E.R.); thais9039@gmail.com (M.T.F.); i.scandagli@gmail.com (I.S.); antonelladicesare@yahoo.it (A.D.C.); federicaricceri@hotmail.it (F.R.); nicola.pimpinelli@unifi.it (N.P.)

**Keywords:** hidradenitis suppurativa, acne inversa, pathogenesis, therapy, cytokines, biologics, obesity, smoking, microbiome, hormones

## Abstract

Hidradenitis suppurativa (HS) is a debilitating, chronic, (auto)inflammatory disease primarily affecting apocrine gland-rich areas of the body. Although pathogenic mechanisms responsible for HS have not yet been fully elucidated, it is a multifactorial process whose main target is the terminal follicle. The role of the inflammatory process (and consequently of cytokine milieu) and of several other factors (genetics, lifestyle, hormonal status, microbiome, innate and adaptive immune systems) involved in HS pathogenesis has been investigated (and often defined) over the years with a view to transferring research results from bench to bedside and describing a unique and universally accepted pathogenetic model. This review will update readers on recent advances in our understanding of HS pathogenesis and novel (potential) medical therapies for patients with moderate-to-severe HS.

## 1. Introduction

Hidradenitis suppurativa (HS), also known as acne inversa, is a debilitating skin disease and may be regarded as a primarily (auto)inflammatory (keratinization) disorder [1]. Researchers have always focused on pathogenetic mechanisms underlying this disease to find novel therapeutic strategies. The evolution of historical terms for HS reveals the understanding of HS pathogenesis over the years [2]. On one hand, “pathogenesis” was found to be the topic of approximately 16% of HS publications from 2008 to 2018, and on the other, “treatment” was the most common publication topic throughout this ten-year period [3].

Thus, in view of these considerations, the aim of this article is to provide the reader with the emerging evidence in HS pathogenesis and an overview of novel potential medical therapies in HS.

## 2. Insights into the Pathogenesis of HS

HS pathogenesis is very complex and puzzling. To date, there is not a universally accepted pathogenetic model that integrates all relevant research findings in a disease overview. Many aspects remain ill-defined; indeed, the time course and causal relationship of pathogenic mechanisms in HS need further investigations: what is the primum movens of HS, inflammation, or follicular occlusion? Is the association with tobacco smoking causal or epidemiologic? Is dysbiosis a consequence or a major player in HS pathogenesis? What is the role of sex hormones and innate immunity? These are just some of the questions still not fully clarified.

### 2.1. How Much Does Genetics Play a Role in HS?

Genetics is essential for providing insight into HS pathogenesis. The role played by genetics in HS is supported by the observation that approximately one third of patients have a positive family history [4]. In 1968, Knaysi and colleagues first described a positive family history for HS in 3 of 18 patients who were specifically asked about it [5]. About 20 years later (first in 1984 and then in 1985), family studies by Fitzsimmons et al. supported the hypothesis of an autosomal dominant inheritance model for familial form of HS [6,7]. These findings were verified by Von der Werth and colleagues in a follow-up study (published in 2000) of the original group observed 15 years earlier by Fitzsimmons [8]. It was only in 2010, that Wang et al., with the aim of investigating the genetic mechanisms underlying HS, discovered γ-secretase gene mutations in a subset of familial HS [9]. The γ-secretase complex is an intramembrane protease composed by 4 different subunits: presenilin, presenilin enhancer-2, nicastrin, and anterior pharynx defective 1, which are encoded by 6 genes, *PSEN1*/*PSEN 2*, *PSENEN*, *NCSTN*, and *APH1A*/*APH1B*, respectively. γ-Secretase is involved in the intramembranous cleavage of numerous type-1 transmembrane proteins, including amyloid precursor protein (APP) and Notch receptors [10]. As of September 2020, 57 mutations of γ-secretase have been reported (in three of the six genes) worldwide: 39 in *NCSTN*, 14 in *PSENEN*, and 4 in *PSEN1* [11]. The downregulation of the Notch signaling pathway secondary to loss-of-function mutations of components of the γ-secretase complex (the intramembrane proteolytic cleavage of the Notch intracellular domain by the γ-secretase complex is essential for signal transduction) has been proposed to play a key role in HS pathogenesis [12]. In contrast to this hypothesis, Frew and Navrazhina demonstrated that no significant downregulation of Notch 1–4 was identified in HS lesional skin, whereas ADAM17 expression was upregulated in HS, in contrast with downregulation seen in other inflammatory dermatoses. In accordance with these findings, ADAM17 (involved in the proteolytic cleavage of γ-secretase substrates, including the extracellular domain of the Notch receptors) could have a potential role in HS pathogenesis [13,14]. Rather recently, Duchatelet et al. have performed mutational analysis of the 6 γ-secretase complex genes in a large cohort of unrelated HS patients: the researchers identified mutations in 8 out of 169 patients, and considering the mutations found in 4 of the 19 patients in their previous studies, the overall prevalence of γ-secretase complex gene mutations was uncommon (12/188; 6.2%) [15]. Jfri and colleagues, in their review, discussed evidence for both monogenic (defects in Notch signaling and in inflammasome function) and polygenic (defects in genes involved in epidermal homeostasis and in inflammation) inheritance of HS [16].

### 2.2. Lifestyle

As often happens in medicine, it is the interaction between environmental factors and genes which leads to the development of a disease. Thus, the role of both obesity and tobacco smoking (although not present in every patient) in HS development is well-accepted and, consequently, HS patients may benefit from a potential lifestyle change [17,18].

#### 2.2.1. Obesity

Several findings have suggested that HS is associated with obesity. Indeed, on one hand, studies in HS patients found that many of these patients are overweight or obese [4,19]. On the other hand, both HS prevalence and severity have increased in obese patients [19,20,21]. A recent population-based analysis in the United States among children and adolescents with HS found that overall obesity prevalence in this group was 68.7%, compared to 29.8% in non-HS pediatric patients [22]. Theut Riis and colleagues even hypothesized that body mass index (BMI) might be used to define two clinically different subtypes: HS patients with a high BMI (BMI > 35) and those with a low BMI (BMI < 25) [23]. Furthermore, a rather recent pooled analysis of six existing case-control studies found a significant association between HS and metabolic syndrome (MetS) [24]. MetS is a cluster of co-occurring (at least three) conditions: dyslipidemia, hyperglycemia, hypertension, and (central) obesity. Sabat et al. showed that MetS (as well as central obesity) was significantly more common in HS patients than in controls (40.0% vs. 13.0%) [25]. Nevertheless, the relationship between HS severity and HS–MetS association remains to be clarified [24,25,26]. Moreover, the prevalence of insulin resistance (a component of MetS) in HS patients was significantly higher compared to age- and sex-matched controls [27]. Obesity may contribute to HS pathogenesis via several mechanisms. Firstly, larger skin folds of obese patients increase (i) mechanical friction, which in turn promotes follicular occlusion and induces rupture of dilated follicles in HS patients, and (ii) temperature and humidity of microenvironment, promoting bacterial proliferation [28,29]. Furthermore, as suggested by Boer and Mihajlovic, patients should be encouraged to wear loose-fitting clothing to avoid friction [30]. In addition, obesity contributes to a systemic low-grade inflammatory state. Indeed, adipocytes and macrophages present in white adipose tissue (a real endocrine organ) secrete “bad adipokines”, which include resistin, chemerin, fetuin-A, and pro-inflammatory cytokines such as tumor necrosis factor alpha (TNF-α), interleukin (IL)-1β, and IL-6. All these molecules may play a role in insulin resistance, alterations of glucose and lipid metabolism, skin inflammation, and vascular and cellular dysfunction [31]. Western diet seems to play a key role in the initial plugging of the follicular duct: dairy products (especially casein, a dairy protein) induce elevated levels of insulin-like growth factor (IGF)-1, whereas whey and simple carbohydrates may raise insulin levels. Consequently, insulin and IGF-1 stimulation make the androgen receptor available to endogenous and exogenous androgens [32]. Furthermore, IGF-1 induces the expression of enzyme 5-α-reductase in the skin [33]. Thus, dairy products and highly refined simple carbohydrates stimulate androgen-driven follicular duct obstruction and subsequent rupture and destruction of the folliculopilosebaceous unit [32]. In their recent systematic review, Sivanand and colleagues reported that dietary interventions (dietary restriction of dairy products and brewer’s yeast) and weight loss may improve HS (except for cases in which weight loss implicates an increase in skin folds and consequently in mechanical stress) [34].

#### 2.2.2. Tobacco Smoking

Smoking habit is common among patients with HS. Konig and colleagues reported that the active cigarette smoker rate was 88.9% in the HS group [35]. Nevertheless, the role of cigarette smoking in HS pathogenesis (epidemiological or causal) has long been a subject of debate among researchers [36]; suffice it to say that even the philosopher of the 19th century Karl Marx (notorious smoker affected by HS) was involved in this debate [37,38]. A rather recent study evaluated the incidence of HS in a heterogeneous population-based sample of about 4 million smokers across the United States, estimating an overall 0.20% HS incidence (during the 3-year study period) among tobacco smokers (vs. 0.11% among non-smokers). Thus, the researchers established that tobacco smoking is associated with an approximately 2-fold increased risk of developing HS and they recommended providing smoking cessation counselling, especially in populations at risk for HS [39]. Nevertheless, in reference to that study, Saleem and colleagues emphasized the weakness of the causal link between HS and smoking; as a matter of fact, they pointed out that 3300 smokers would be needed to have 1 additional HS case attributable to smoking over a year, concluding, thus, that smoking rarely causes HS [40]. According to Micheletti, in an attempt to understand whether smokers are at greater risk of developing HS or whether smoking is a result of this chronic disease (often associated with anxiety and depression), the retrospective study by Garg et al. [39] (albeit with some limitations) better defines the link between smoking and HS, highlighting the important role of clinicians in smoking cessation [41]. The association of tobacco smoking with HS severity was also investigated. Dessinioti and colleagues (similar findings were reported by Matusiak and co-workers) found that HS severity according to Hurley staging was not associated with smoking habit (current or former); nevertheless, they showed that smokers (current or former), in comparison with non-smokers, were at increased risk of having more than two body areas affected, confirming a correlation between smoking habit and the number of body areas impacted by the HS lesions [42,43]. Notwithstanding that the role of smoking in HS pathogenesis is not entirely clear, there is increasing evidence suggesting that smoking status may influence treatment response. Indeed, Kromann et al. observed that 66% (33/50) of HS patients who reported disease remission were non-smokers (vs. 34% of active smokers), suggesting that non-smoking status is linked to a greater rate of HS self-reported remission [44]. According to these findings, Denny and colleagues found that non/former smokers were more likely to have HS improvement in response to first-line medical therapy (including both topical and systemic antibiotics and intralesional corticosteroids) compared with current smokers [45]. Evaluating how smoking may affect HS lesions has proved useful for better understanding the (potential) relationship between smoking and HS. HS is characterized by an increased T helper 17 (Th17) cell/regulatory T (Treg) cell ratio (see below), and smoking may aggravate this ratio. Indeed, agonists contained in cigarette smoke (such as benzopyrene) induce a rise of Th17 cells (and consequently of IL-17 expression) and a decrease of Tregs via activation of the aryl hydrocarbon receptor (AhR) signaling. In addition, Notch-related gene expression (whose role is described above) was downregulated in smokers [46]. Nicotine, a component of tobacco, may increase intracellular cyclic adenosine monophosphate (cAMP) levels, strengthening IL-10 production by monocytic cells [47]. Nicotine may influence epidermal morphology, inducing a significant epidermal hyperplasia [48]. Furthermore, nicotine may induce dysbiosis and promote the biofilm formation [49]. Nowadays, effects of “vaping” on HS should be investigated, as given that e-cigarette (considered a safer option than conventional cigarettes) solution is often composed of nicotine, traditional and e-cigarettes may partially share the smoking effects described above [50].

### 2.3. The Role of Cutaneous Microbiome in HS

Skin “microbiota” (the whole and complex community of bacteria, viruses, and fungi which inhabit the skin surface, the hair follicles, and the dermis) and/or “microbiome” (the set of microorganisms, their genomes, and the surrounding environmental habitat) research investigates the composition (and diversity) of human skin microbial communities at different sites. The impact of these microbes on pathogenesis of skin diseases, including HS, is an important aim for researchers [51]. Skin topography influences microbial colonization and composition; in the apocrine gland-rich skin (moist skin), modification of the homeostatic symbiosis between microbiota and cutaneous immune system (both specific for these skin regions) might lead to (IL-17 type) inflammation in HS [52]. Several findings suggested that HS is associated, as other inflammatory skin diseases (including atopic dermatitis and acne vulgaris), with dysbiosis, that is an imbalance (any change to the composition) of the skin “healthy” resident microbiome. Indeed, microbial dysbiosis in HS has been the subject of over 200 publications; nevertheless, its exact role in HS pathogenesis remains elusive and is yet to be fully clarified [53]. In their recent review (which includes 21 studies examining the cutaneous microbiome), Wark and Cains reported that (i) *Staphylococcus aureus*, coagulase-negative staphylococci, and Enterobacteriaceae species were commonly cultured from HS skin, and (ii) *Porphyromonas* and *Prevotella* (both anaerobic bacteria) were relatively increased in HS skin, as demonstrated by 16S rRNA sequencing studies [54]. In their case-control study, Ring and colleagues, using next-generation sequencing (NGS) to explore the follicular skin microbiome of HS patients and healthy controls, identified 5 microbiome types and demonstrated that microbiome differs significantly in HS (lesional and non-lesional) patients compared with that in healthy controls. The researchers found that (i) *Corynebacterium* species (type I) or *Porphyromonas* and *Peptoniphilus* species (type IV, which is not revealed in healthy controls) were predominant in lesional skin; (ii) conversely, *Acinetobacter* and *Moraxella* species (type II) were prevalent in non-lesional skin, and (iii) *Propionibacterium acnes* (type V; *P. acnes* has recently been renamed *Cutibacterium acnes* [55]) showed a significantly higher relative abundance in healthy controls compared with HS skin [56]. More recently, Ring et al., using NGS to investigate the microbiome of tunnels, found that *Porphyromonas* spp. and *Prevotella* spp. (and *Corynebacterium* spp.) were the most frequent genera in HS tunnels. Furthermore, the researchers, in accordance with previous findings [56], did not reveal *Propionibacterium* spp. in the most abundant genera found in HS tunnels, and thus, they hypothesized that this deficiency (of *Propionibacterium* spp.) might lead to a skin dysbiosis in HS [57]. Dysbiosis may contribute to HS pathogenesis via several mechanisms. Indeed, skin microbes may (i) directly produce and control antimicrobial peptides and proteins (AMPs) production by keratinocytes, (ii) activate C3a and C5a pathways (see below), (iii) control expression level of IL-1, and (iv) increase the production of cytokines such as IL-17A and interferon- γ (IFN-γ) by dermal T cells [58,59,60,61]. Interestingly, *Porphyromonas* and *Prevotella* may contribute to HS pathogenesis, promoting excessive AMP secretion, and *Prevotella* has been shown to lead to production of IL-23 and IL-1 [54,62,63]. The risk of superinfections is more prevalent in severe patients in which bacteria may contribute to maintain chronic inflammation [64]. Further microbiological studies could clarify whether the lymph node involvement in HS is due exclusively to inflammatory cell infiltration or also to secondary bacterial infection [65]. Recently, Yidana has hypothesized that fungi have a potential role in HS pathogenesis through the Ahr activation [66]. Despite numerous studies conducted, to date, it remains to be clarified whether dysbiosis represents a possible trigger of the HS pathogenetic process or is a consequence of the underlying inflammatory process [54].

### 2.4. Sex Hormones

The link between sex hormones and HS remains elusive; nevertheless, Hurley suggested a role for hormones in HS pathogenesis as early as 1979. Important clues about the potential role of sex hormones in the pathogenesis of the disease include (i) gender (female) prevalence of HS, (ii) age of onset (HS is rare both prior to puberty and after menopause), (iii) premenstrual HS flares, (iv) HS severity changes during pregnancy, and (v) efficacy of antiandrogen therapy on HS [67,68,69]. HS usually occurs between the ages of 11 and 30, the age range in which cutaneous androgen enzyme activity is at its maximum. Premenstrual flares are common in women with HS (studies found an occurrence of 44–63%) [68]. Recently, Collier and colleagues confirmed HS worsening with menses (62.4% of 282 women), mainly during the week preceding their onset (138 of 175 women) [70]. Concerning the impact of pregnancy on HS, an amelioration of HS during pregnancy and a deterioration post-partum were reported in the literature [68]. According to Vossen et al., this amelioration was found more frequently in those women who reported perimenstrual deterioration of HS symptoms compared to those whose symptoms improved or were unaffected during menses [71]. Pregnancy may affect women with HS in several ways: (i) progesterone, whose serum concentrations are increased in pregnancy (and decreased during the week preceding the onset of menses), inhibits Th17 cell differentiation, (ii) IL-1 receptor antagonists (IL-1RA) and soluble TNF-α receptor (TNF-R) increase in late pregnancy, neutralizing the effects of both IL-1 and TNF-α respectively, and (iii) on the other hand, metabolic dysregulation (and weight gain) during pregnancy might worsen HS symptoms [72]. Starting from similarities/differences between HS and acne vulgaris (a well-known androgen-dependent disorder), several studies investigated circulating hormones (especially androgens) in HS [68,69]. Harrison and colleagues described the hormonal background of HS patients first. The researchers found no significant differences in serum mean basal levels of testosterone, dehydroepiandrosterone sulphate (DHEA-S), progesterone, estradiol 17β, free T3, free T4, prolactin (PRL), thyrotrophin (TSH), luteinizing hormone (LH), and follicle-stimulating hormone (FSH) of 13 women with HS compared with 9 age-matched female volunteers (all patients and controls were tested in the luteal phase) [73]. According to these findings, Jemec, in 1988, showed that the incidence of women with signs of androgenization did not differ from age-matched healthy controls [74]. Conversely, Mortimer et al. reported a higher concentration of total testosterone and free androgen index (FAI, the ratio of total testosterone/sexual hormone-binding protein (SHBG) concentrations) in HS patients compared to age-matched controls without cutaneous signs of virilization (a higher concentration was maintained even after the exclusion of HS patients with coexistent hirsutism or acne). These findings led the authors to hypothesize an androgenic basis for HS that is supported by alterations in plasma levels of available androgens and/or an excessive rate of peripheral conversion of androgens by the apocrine glands [67]. Interestingly, Harrison and colleagues reported significantly higher androgen (testosterone, androstenedione, and the androgen index) serum levels and lower progesterone levels in those patients without a premenstrual flare, supporting the hypothesis of an enhanced peripheral androgen conversion in those patients with a premenstrual flare and concomitant normal serum androgen levels [75]. Nevertheless, Barth et el., investigating the androgen metabolism in apocrine glands of HS patients and age-matched controls, found a significantly lower activity of 3 β-hydroxysteroid dehydrogenase (β-HSD) and 17 β-HSD (enzymes that catalyze the production of progesterone and testosterone, respectively) in HS compared with controls, and a similar 5-α reductase activity in both groups. Thus, they concluded that exaggerated activities of end-organ androgen interconverting enzymes cannot be related to HS [76]. Buimer and colleagues, considering the anatomical distribution of HS in apocrine gland-rich skin and the possible relation between sex hormones and occurrence of HS, performed an immunohistochemical analysis of steroid hormone receptors in HS patients. The researchers found no significant difference in the expression of androgen receptor (AR) and estrogen receptor (ER) in apocrine glands in HS compared with healthy skin [77]; nevertheless, an increased post-receptor response to androgens cannot be ruled out [68]. Rather recently, Gaunter, in his transcriptomic analysis from skin biopsies of 17 HS patients and 13 patient-matched controls, showed an increased AR transcriptional activity in HS lesions and an upregulation of genes under the transcriptional control of three key stem cell transcription factors. Thus, the author suggested that hormonal dysregulation of epidermal stem cells (resident within an androgen-regulated structure, the bulge, of the pilosebaceous unit) may play a role in keratinocyte proliferation and differentiation, and consequently in follicular plugging [78]. A whole transcriptome profiling from lesional skin of HS patients revealed that the gene upregulation profile of female apocrine glands was related to the androgen signaling pathway, supporting the hypothesis of an androgen regulation of apocrine glands in female lesional skin [79]. The role of antiandrogen treatment, as an alternative or concomitant therapy, still remains to be defined [80].

### 2.5. The Histopathological Findings

Terminal follicle is the main histopathological target in HS [81,82,83]. Von Laffert and colleagues investigated very early lesions at terminal follicles (and surrounding tissue). Follicular hyperkeratosis, hyperplasia of follicular epithelium, and perifolliculitis, the major histopathological features of HS, were observed in 82% (77/94), 77% (72/94), and 68% (64/94) of samples, respectively. Conversely, ruptures of the follicle structure were only evident in 28% (26/94) of tissue samples. Furthermore, morphological evaluation revealed an epidermal psoriasiform hyperplasia with a subepidermal cellular inflammatory infiltrate at interfollicular epidermis. Thus, the researchers concluded that the rupture of the follicle is preceded by perifolliculitis and follicular hyperkeratosis (both early events). Cellular infiltrate (both perifollicular and subepidermal) was composed of CD-3, CD-4, CD-8, CD-68, and CD-79 cells, and the authors reported a CD8 cell epitheliotropism (follicular and epidermal). The inflammatory process (especially the cytokine milieu) might drive keratinocyte proliferation both in the follicular epithelium and interfollicular epidermis [84]. The authors, further investigating the chronology of early lesions in HS, (i) confirmed the prevalence of histopathological findings described above and (ii) hypothesized that (infundibular) follicular epithelial hyperplasia of the terminal hair (probably the origin of sinus tracts) is an early event that precedes the rupture of the follicle (a secondary event). Thus, they concluded that a bilocated (inflammation-driven) epithelial hyperplasia is present in HS [85]. Danby et al. showed that the (PAS-positive) basement membrane zone was altered (missing, wispy, or fragmented) in the folliculopilosebaceous unit (especially at the sebofollicular junction) of patients with HS. Hereby, the researchers hypothesized that this alteration might implicate the release of both material able to trigger inflammatory processes and free-living stem cells (at the origin of epithelialized sinuses) [86]. Histological examinations suggested that apocrine sweat gland involvement (the term HS originates from the hypothesis that apocrine glands were the focus of the pathogenetic process) is a secondary phenomenon [81,82,85]. Rather recently, Zouboulis and colleagues, in their whole transcriptome profiling study, have confirmed that apocrine glands are bystanders in HS, with a gender-specific involvement [79]. Sebaceous glands are small or absent. Abscess and draining sinus tract (lined by stratified squamous epithelium) formation represents an advanced event [87]. CD68+ macrophages are the most abundant infiltrating cells found in the dermis of HS lesions [88]. They are a major source of numerous pro-inflammatory cytokines (including IL-23, IL-1β, and TNF-α) and their dysfunction (to which obesity and smoking contribute) may have a critical role in HS pathogenesis [89]. B cells’ role in HS is still unclear and it is unlikely that they are primarily pathogenic. Regardless, as reported by Frew et al., (i) tertiary lymphoid organs (TLOs), where autoreactive B cells develop and interact with T cells, have been identified in HS, (ii) an upregulation of immunoglobulin (Ig)G3/IgG1, kappa light chains, lambda light chains, IgD, and IgA1 in lesional HS was demonstrated, and (iii) B cells may amplify the inflammatory response and contribute to the fibrotic process (via IL-6 and transforming growth factor-β, TGF-β) in HS lesions [90]. Fibroblasts might play a key role in scarring and tunnels’ formation in HS [91]. Infiltration of neutrophils (and the presence of neutrophil extracellular traps, NETs) was confirmed in HS skin lesions when compared to skin from healthy donors [92]. The granulocyte colony-stimulating factor (G-CSF), whose major inducers are interleukin IL-1β and IL-17, is a key regulator of neutrophil biology; recently, Wolk and colleagues demonstrated, at both the mRNA and protein levels, for the first time, highly increased levels of G-CSF in HS lesional skin. In addition, researcher analysis revealed that G-CSF upregulates several molecules (abundant in HS lesions) in neutrophils, such as Matrix Metalloproteinase (MMP)8, MMP9, MMP25, and ADAM8 (disintegrin and metalloproteinase domain-containing protein 8) [93].

### 2.6. Innate Immunity

HS is characterized by alterations of the innate immune system [94]. AMPs are a group of ancient, generally small, effector molecules of the innate immune response. The AMP family comprises several types of peptides, such as human β-defensins (hBD) 1–3, cathelicidin LL-37, ribonuclease RNase-7, S100 calcium binding protein A7 (S100A7, also known as psoriasin), S100A8 (calgranulin-A), S100A9 (calgranulin-B), S100A7A (also known as S100A15 or koebnerisin), and dermcidin. They are produced by different resident skin cells, and they carry out numerous roles in human skin, including antimicrobial activity, modulation of inflammatory responses, maintenance of skin barrier homoeostasis, and wound-healing activity [95]. Transcriptome studies suggested a significant role of AMPs in HS pathogenesis. This idea was confirmed by Wolk and colleagues, who found, on one hand, an upregulation of hBD2, hBD3 (but not hBD1), S100A7, S100A8, and S100A9 in HS lesions compared with healthy skin, and on the other hand, a relative deficiency of AMP levels in HS skin compared to those found in psoriasis lesions (hBD1, hBD3, and S100A7 expression in HS lesions was even lower than that in atopic dermatitis lesions) [96]. According to these findings, a relative significant reduction of both hBD2 and hBD3 in HS compared with psoriasis and atopic dermatitis skin was recently found [97]. Hofmann et al. found that expression of hBD3 was reduced in severe HS (Hurley grade III) and that of RNase-7 was lower in lesional skin of HS patients, across all severity stages of the disease [98]. AMP expression analysis of keratinocytes isolated from hair follicles showed that S100A7, RNase-7, and S100A8 were increased by 9-, 7-, and 5-fold in HS compared with healthy donors, respectively. In contrast, hBD1 gene expression in HS was decreased by 3-fold when compared to healthy donor hair follicle cells [99]. Bechara and colleagues also demonstrated a significant increase in mRNA expression of both hBD2 (active preferentially against Gram-negative bacteria) and LL-37 (active against a variety of Gram-negative bacteria, Gram-positive bacteria, and Candida species) in HS lesions compared to non-lesional skin [100]. The human cathelicidin LL-37 is produced by keratinocytes, neutrophils, macrophages, T cells, and B cells. Thomi et al. confirmed that it was upregulated at the peptide level in lesional HS skin and, in an interesting way, they showed that LL-37 peptide levels in lesional HS skin positively correlated with both the presence of T cells, macrophages, and neutrophils, and mRNA levels of the Th1/Th17-associated cytokines IFN-γ, IL-17, IL-23, IL-1β, TNF-α, and IL-32 [101]. Taking into consideration an anionic AMP, Shanmugam and colleagues showed that dermcidin, a sweat gland-associated AMP (produced by eccrine sweat gland and sweat duct epithelial cells), was significantly downregulated in HS skin compared to healthy donor skin [102]. In accordance with these findings, Coates et al. confirmed the significant decrease (of nearly 12-fold) in the expression of dermcidin in HS lesional samples (whose expression is instead increased in wounded skin) compared with non-lesional skin. In addition, the researchers found that S100A7, S100A8, and S100A7A expression was significantly increased in HS lesional skin samples, suggesting that changes in AMP expression and altered sweat gland function might drive HS pathogenesis [103]. Rather recently, Wolk and colleagues have found that lipocalin-2 (LCN2) was significantly upregulated in the serum (and highly expressed in lesional skin) of HS patients compared to healthy participants, demonstrating a positive correlation between LCN2 blood levels and disease activity (Sartorius score). LCN2 is a multifunctional glycoprotein secreted by activated granulocytes and (to a lower extent) keratinocytes of HS lesions, and according to the authors, it might be used as a blood biomarker [104].

The complement cascade, which represents a major component of the innate immunity, may be activated via three different (interconnected) mechanisms: the classical, lectin, and alternative pathways. All three pathways generate C3 convertase, which drives the production of C5 convertase and, eventually, of the membrane attack complex (MAC) C5b-9. It is not yet clear whether complement dysregulation is a primum movens in the HS pathogenesis or a consequence of advanced stages of disease [105]. Some bacterial species (found in HS microbiome) may activate C3a and C5a pathways, which in turn may be involved in the nucleotide-binding oligomerization domain (NOD)-, leucine-rich repeat (LRR)-, and pyrin domain (PYD)-containing protein 3 (NLRP3) activation [58]. Kanni et al. demonstrated a high systemic activation of complement in HS patients whose plasma concentrations of C5a and C5b-9 were significantly higher than controls and greater among patients at Hurley stage I than Hurley stages II and III patients. Furthermore, stimulated peripheral blood mononuclear cells (PBMCs) of HS patients, with the addition of 25% of the patient’s plasma, overproduced TNF-α. This result was attenuated by the addition of IFX-1 (a C5a blocker), thus supporting the hypothesis that C5a is a mandatory element for TNF-α production by this cell subset [106].

### 2.7. HS Cytokine Milieu

To investigate the cytokine profile in lesional, perilesional, and uninvolved skin, as well as in exudate and serum of HS patients, has been, is, and will continue to be, crucial to fully understanding HS pathogenesis [107]. Undoubtedly, cytokines act like a biological alphabet for the communication between cells both in physiologic and pathologic processes. It is interesting to note that Ardon and colleagues firstly used a Transdermal Analysis Patch (TAP, FibroTx, Tallinn, Estonia), a non-invasive diagnostic tool (vs. invasive techniques such as biopsies), for assessing the expression of cytokines in HS skin [108].

#### 2.7.1. IL-1 Cytokine Family

The IL-1 family includes a total of 11 members: 7 pro-inflammatory agonists (IL-1α, IL-1β, IL-18, IL-33, IL-36α, IL-36β, and IL-36γ), 3 receptor antagonists (IL-1Ra, IL-36Ra, and IL-38), and 1 anti-inflammatory cytokine (IL-37) [109]. The two major IL-1 proteins, IL-1α and IL-1β, share the same biological functions and bind to the same receptor, IL-1 type 1 receptor (IL-1R1). IL-1β is mainly released by monocytes and macrophages (and it circulates systematically); nevertheless, dendritic cells (DCs), neutrophils, B and T cells, endothelial cells, epithelial cells, mast cells, and dying cells are other common cellular sources of IL-1 [109,110]. Kelly et al. demonstrated that CD11c+ CD1a- CD14+ cells were the main source of IL-1β in HS patient skin. These cells, previously identified in the skin as an additional DC subset (CD14+ dermal DC subset), are a population of monocyte-derived macrophages with a short half-life [111,112]. IL-1β (influencing every cell type) induces the production of neutrophil-attracting chemokines (chemokine (C-X-C motif) ligand 1 CXCL1, CXCL6, and CXCL8) and MMPs (MMP1, MMP3, MMP10), mediating the influx of neutrophils into the damaged site [47]. IL-1α (that acts locally) is expressed by many cell types, including endothelial cells and keratinocytes [110]. High concentrations of both IL-1α and IL-1β have been found in the pus derived from lesions of HS patients [113]. The IL-1 pathway resulted hyperactive in HS, and van der Zee and colleagues showed not only that IL-1β levels were significantly elevated both in lesional and perilesional HS skin, but that they were also higher than in psoriasis skin (the prototype inflammatory skin disease) [114]. In accordance with these findings, more recently, Witte-Händel et al., examining the cytokine profile of HS vs. psoriasis skin lesions, found that IL-1β levels in HS lesions exceeded those in lesional psoriatic skin by approximately 8-fold [115]. The NLRP3 inflammasome is a cytoplasmic high-molecular-weight protein platform that plays a key role in IL-1β maturation and secretion from cells. The NLRP3 inflammasome consists of NLRP3 (the sensor), ASC (the adaptor), and caspase 1 (the effector). It is activated in response to the recognition of a variety of pathogen-associated molecular patterns (PAMPs) and/or damage-associated molecular patterns (DAMPs) [109,116]. Thus, to investigate the role of the NLRP3-caspase 1 pathway in HS pathogenesis, Kelly and colleagues found that caspase 1 activation was enhanced in HS skin and was associated with a higher expression (in HS lesional skin compared with healthy control samples) of both NLRP3 and IL-18 (a caspase 1-dependent pro-inflammatory cytokine) [111]. Lima et al. confirmed that both NLRP3 and caspase 1 were upregulated in lesional epidermis of HS, demonstrating by Western blot analysis that caspase-1 p10, the active form of caspase 1, resulted significantly increased in keratinocytes obtained from lesional skin as compared with perilesional skin [117]. Accumulation of reactive oxygen species (ROS) plays a key role in both priming (signal 1) and activation (signal 2) of NLRP3 inflammasome, and NADPH oxidase (NOX) enzyme complexes are the main source of endogenous ROS. Nevertheless, Frings and colleagues excluded that altered NOX enzymes’ mRNA expression accounts for NLRP3 inflammasome activation in HS patients [118].

The expression levels of IL-36α, IL-36β, and IL-36γ resulted significantly increased in lesional HS skin compared to healthy controls, while IL-36Ra (the natural anti-inflammatory receptor antagonist) expression in HS tissue samples was variable [119,120,121]. Hassam et al. proposed that IL-36α was involved in the later stages of the HS inflammatory process since it was greatly expressed in lesional HS skin (IL-36α was expressed in 27% of the healthy controls samples, 27% of the perilesional HS skin samples, and 87% of the lesional HS skin samples). Vice versa, IL-36β (aberrantly expressed in HS perilesional skin) might be involved in the early steps of the pathophysiology process. IL-36 cytokines were found mainly in keratinocytes, as revealed by immunohistochemical analysis. This latter finding, together with the increased expression of IL-36 cytokines in lesional HS skin, led the researchers to hypothesize the existence of an autocrine loop in HS, maintained by the simultaneous secretion of IL-36 and the expression of IL-36 receptors (IL-36R) by both keratinocytes and dendritic cells (a major source of proinflammatory cytokines) [121]. According to Thomi and colleagues, the lower IL-36 levels in HS compared to psoriasis skin could be due to psoriasis hyper-keratinization, since keratinocytes are the major producers of IL-36 [120]. To investigate IL-36 systemic levels in HS patients, Hayran et al. showed that serum levels of IL-36α, IL-36β, and IL-36γ were significantly elevated (this increase was not dependent on smoking, obesity, and MetS) compared to healthy controls and positively correlated with systemic inflammatory parameters (such as high-sensitivity C-reactive protein (hsCRP), CRP, and erythrocyte sedimentation rate (ESR)) [122].

#### 2.7.2. TNF-α and IFNs

The efficacy of anti-TNF-α treatments (adalimumab and infliximab monoclonal antibodies) suggests an important role of TNF-α in HS pathogenesis [123]. Rather recently, multiple studies on the possible mechanisms underlying the therapeutic effects of anti-TNF-α molecules in HS have been carried out. Moran and colleagues have showed the effects of anti-TNF-α therapy in skin-infiltrating T cells: a significant reduction of Th17 cells’ frequency was seen in both the lesional and perilesional skin of HS patients treated with anti-TNF-α compared with anti-TNF-α-naïve patients, and the Th17:Treg cell ratio (this ratio was significantly increased in HS lesional skin compared with healthy control samples) was normalized [124]. After 36 weeks of adalimumab therapy, Th17 lymphocyte- and neutrophil-related inflammatory serum markers (IL-1β, IL-6, IL-8, IL-10, IL-17A, soluble TNF receptor I (sTNF-RI), sTNF-RII, CRP, and ESR) decreased significantly in HS patients [125]. Balato et al. demonstrated that the mammalian target of rapamycin complex 1 (mTORC1) pathway (upregulated in HS lesions) is modulated by anti-TNF-α therapy [126]. In support of these findings, efficacy of sirolimus (a potent mTOR inhibitor) in combination with TNF-α blockade for severe HS was confirmed in a small retrospective study [127]. TNF-α is a proinflammatory cytokine and, although produced by many different cell types, macrophages and monocytes represent its main source. TNF-α has pleiotropic effects on different cell types: it induces a series of various inflammatory molecules, including cytokines and immune-cell-attracting chemokines, and contributes to endothelial activation [47]. Surely, activation of TNF-RI, which is expressed on keratinocytes, is responsible for the expression of proinflammatory cytokines (IL-1 and IL-6), chemokines (CXCL8 and chemokine (C-C motif) ligand 20, CCL20), and adhesion molecules (intercellular adhesion molecule 1, ICAM-1). Furthermore, immune response in keratinocytes is amplified by the synergistic effect with other cytokines, such as IL-17A and IL-17C [128]. TNF-α resulted as the sole cytokine that induced LCN2 in granulocytes and, in cooperation with IL-17, in keratinocytes [104]. TNF-α expression, at both the mRNA and protein levels, was significantly higher both in lesional and perilesional HS skin than in healthy control samples (with no significant differences between lesional and perilesional HS skin) [111,114]. TNF-α levels in HS skin were higher (5-fold) compared to those in psoriasis and they tended to correlate with disease severity [114]. Mozeika and colleagues confirmed a wide distribution of TNF-α-positive cells in skin from patients with HS (TNF-α-positive cells were not present in healthy skin) [129]. Dréno et al. demonstrated a significant decrease in the expression of TNF-α in lesional and non-lesional skin of HS patients compared with control skin [130].

IFN-γ is a pleiotropic soluble cytokine, the unique member of the type II IFN family. It is secreted predominantly by natural killer (NK) cells, CD4+ T helper type 1 (Th1) cells, and CD8 cytotoxic T cells. IFN-γ (originally identified as macrophage activation factor) is a major activator of macrophages [131]. IFN-γ levels were significantly elevated in the HS wound effluent compared to age-matched patients with chronic wounds [132]. Gudjonsson and colleagues confirmed that IFN-γ (and IL-36) responses are dominant keratinocyte immune responses in HS [133]. On the other hand, Byrd et al. proposed that upregulation of the type I IFN pathway in HS skin might be related to IFN-α-secreting-plasmacytoid DCs (pDCs), in turn activated by NETs [92].

#### 2.7.3. IL-17 Cytokine Family

The IL-17 family consists of 6 structurally related cytokines, IL-17A through IL-17F. IL-17A was first cloned in 1993 [134]. IL-17A and IL-17F (in the form of homodimers or heterodimers) are secreted by activated lymphocytes, and they are produced mainly by Th17 cells, but also by other cell populations, such as CD8 T cells (Tc17), gamma delta (γδ) T cells, group 3 innate lymphoid cells (ILC3), and invariant natural killer (iNK) T cells [134,135]. Th17 cells (a CD4+ T helper subset discovered in 2005) differentiate from naïve T cells in response to cytokines, including TGF-β, IL-1β, IL-6, and IL-23 (essential for final maturation of Th17 cells). DCs alone are sufficient to promote Th17 cell differentiation and activation because they simultaneously present antigens and may provide the differentiation factors [134,136]. Homodimers and heterodimers of IL-17A and IL-17F bind to their heteromeric receptor complex, which is composed of IL-17RA (firstly described in 1995) and IL-17RC subunits. IL-17C and IL-17E (IL-25) are expressed mainly by keratinocytes. IL-17C and IL-17E transduce signals through the IL-17RA/IL-17RE and IL-17RA/IL-17RB receptor complexes, respectively [134,135]. Both IL-17C and IL-17A stimulate keratinocytes (inducing epidermal hyperproliferation) with strong induction of AMPs, including S100A7-A9 proteins and cytokines or chemokines (such as CXCL1, IL-1, IL-8, CCL20, and IL-36). IL-17A and IL-17C are both involved in potentiating the inflammatory loop: on one hand, IL-17A induces the secretion of IL-17C in keratinocytes, while on the other hand, IL-17C may stimulate Th17 cells to increase synthesis of IL-17A/F (and IL-22) [137]. When Witte-Händel and colleagues examined cytokine milieu in HS skin lesions, IL-17A and IL-17F reached levels comparable with those observed in psoriasis lesions; in addition, IL-17A strengthened the action of IL-1β in keratinocytes [115]. Indeed, IL-1β and IL-17 could be involved in a positive feedback loop: on one hand, IL-17 (and IL-22), whose receptors are constitutively expressed on keratinocytes, stimulates IL-1β secretion by keratinocytes via activation of the ROS-NLRP3-caspase-1 pathway, on the other hand, Th17 cells (which possess IL-1β receptors) are stimulated by IL-1β to generate IL-17A and IL-17F (and IL-22) [138]. Furthermore, Lima et al. showed a significantly increased number of IL-17-positive cells in both lesional and perilesional HS skin when compared with healthy controls, with no difference in the number of IL-17-positive cells between perilesional and lesional HS skin. The researchers observed a high number of IL-17-expressing neutrophils in the deep infiltrate (in contrast to a considerably lower number of cells classified as Th17 cells), and they concluded that infiltrating neutrophils were a source of IL-17 [117]. Navrazhina and co-workers showed a significant elevation of mRNA levels of IL-17C in lesional, perilesional, and unaffected HS skin compared with healthy controls and comparable to those observed in psoriasis skin [139]. In addition, Matusiak and colleagues reported increased IL-17 serum concentrations in patients with HS [140]. According to these findings, IL-17A serum levels showed a significant linear correlation with clinical inflammatory activity in HS patients [141]. Vice versa, the study of Öztürk found no significant difference in IL-17A (and TNF-α, IL-1β, IL-23) serum levels compared to controls and post-treatment levels [142].

#### 2.7.4. IL-12 Cytokine Family

The IL-12 cytokine family includes four members, IL-12, IL-23, IL-27, and IL-35. IL-12 (discovered in 1989) is secreted by innate cells, including macrophages and DCs, and a combination of IL-12 and IFN-γ (produced by natural killer cells) drive the differentiation of Th1 cells from naïve T cells. IL-12 is a heterodimeric cytokine composed of the IL-12p40 and the IL-12p35 subunits. IL-23 (discovered in 2000) is produced by DCs and macrophages, and it is composed of IL-12p40 and IL-23p19 subunits. As noted above, its role is essential to guarantee final maturation of Th17 cells (the so-called IL-23/Th17 axis) [136,143]. Schlapbach and colleagues showed that both the IL-12/Th1 and the IL-23/Th17 pathways are expressed in HS. Indeed, the researchers found that both IL-12 and IL-23 were abundantly expressed (compared to healthy control subjects) by activated macrophages infiltrating the papillary and reticular dermis in HS lesions [144]. According to these findings, Vossen et al. demonstrated that IL-12/23p40 expression was significantly higher in the skin of HS patients compared with healthy controls [145].

#### 2.7.5. IL-10 Cytokine Family

The IL-10 cytokine family consists of a total of nine members, IL-10, IL-20 subfamily members IL-19, IL-20, IL-22, IL-24, and IL-26, and the related cytokines IL-28A, IL-28B, and IL-29, which are designated as IFN-λ2, IFN-λ3, and IFN-λ1, respectively [146]. A relative deficiency of IL-22 and IL-20 levels (although increased compared with healthy skin), but not of IL-17A, IL-26, IFNγ, IL-24, or IL-1β, was found in HS lesions compared with psoriasis lesions. IL-22, which in turn induces IL-20 in keratinocytes, may be produced by almost all lymphocyte subsets, especially by Th17 and Th22 cells [96,146]. Wolk and colleagues demonstrated that this relative deficiency in IL-22 expression was not caused by reduced T cell infiltration but was the result of IL-10 (whose levels negatively correlated with IL-22 expression), highly expressed in HS lesions. Furthermore, the researchers found that IL-22 and IL-20 were important regulators of keratinocyte AMP expression [96]. Indeed, IL-22 acts in part via stimulation of AMPs through a STAT3-dependent pathway. Jones et al., using an in vitro scratch assay, demonstrated that IL-22 amounts produced by HS (defective) keratinocytes were significantly lower compared to normal and chronic wound keratinocytes, suggesting a crucial role of IL-22 signaling in HS pathogenesis [147]. Van der Zee and colleagues confirmed that levels of IL-10 were significantly higher both in lesional and perilesional HS skin than in healthy control skin [114]. IL-10 production was increased 15-fold in HS skin compared with healthy control subjects [148].

Scala et al. attempted to explore the role of IL-26 (an antimicrobial and pro-inflammatory cytokine expressed by Th17 cells) in HS. First, IL-26 plasma levels were significantly increased in HS patients compared to healthy control subjects, and this increment was significantly correlated with disease severity. Second, IL-26 expression was significantly higher in HS (as well as in psoriasis and atopic dermatitis) lesional skin compared to skin of healthy control subjects, and its inhibition significantly reduced both hBD2 and hBD3 gene expression. Third, PBMC antimicrobial activity, cytotoxicity, and killing activity against *S. aureus* were lower in HS patients compared to healthy subjects. All these findings allowed researchers to hypothesize the existence of a link between skin antimicrobial incompetence in HS and IL-26 [97].

#### 2.7.6. IL-6

IL-6 is a pleiotropic cytokine with both pro-inflammatory and anti-inflammatory roles. It induces the production of acute phase proteins, promotes synthesis and secretion of various Igs, and in combination with TGF- β, IL-1β, and IL-23, differentiation from naïve T cells [149]. Studies have reported controversial results on the association between IL-6 and HS. The mRNA expression of IL-6 was significantly higher in HS compared to non-lesional skin [100]. On the contrary, Dréno and colleagues observed significantly decreased IL-6 expression in non-lesional and lesional HS skin [130]. Xu et al. found that serum IL-6 levels were significantly increased in (i) Hurley II and III HS patients compared to a healthy control group, and (ii) patients with lesions covering >5% of their body surface compared to those with lesions covering <5% [150].

### 2.8. An Overview of the Pathogenetic Mechanisms in HS

**Figure 1:**
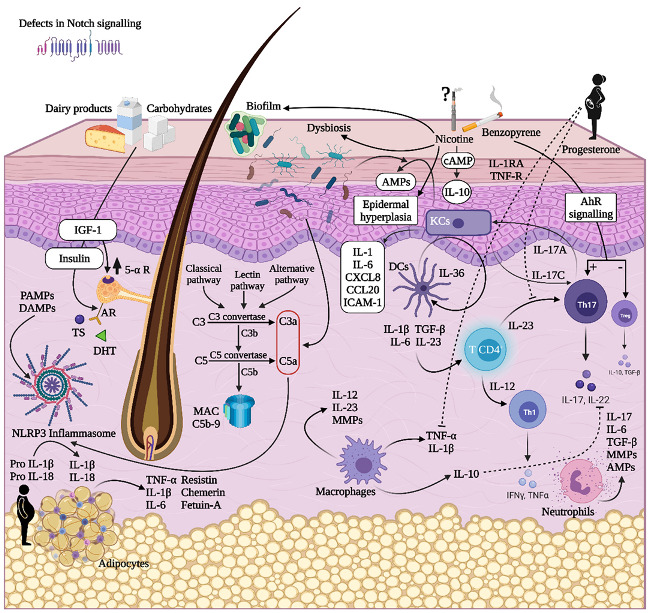
The main pathogenetic events in HS. Created with BioRender.com. Abbreviations: IGF-1, insulin-like growth factor 1; AR, androgen receptor; TS, testosterone; DHT, dihydrotestosterone; 5-α R, 5-α-reductase; PAMPs, pathogen-associated molecular patterns; DAMPs, damage-associated molecular patterns; NLRP3, nucleotide-binding oligomerization domain (NOD)-, leucine-rich repeat (LRR)-, and pyrin domain (PYD)-containing protein 3; TNF-α, tumor necrosis factor alpha; IL, interleukin; MAC, membrane attack complex; MMPs, matrix metalloproteinases; TGF-β, transforming growth factor-β; AMPs, antimicrobial peptides and proteins; CXCL8, chemokine (C-X-C motif) ligand 8; CCL20, chemokine (C-C motif) ligand 20; ICAM-1, intercellular adhesion molecule 1; cAMP, cyclic adenosine monophosphate; AhR, aryl hydrocarbon receptor; KCs, keratinocytes; DCs, dendritic cells; IFN-γ, interferon-γ.

A schematic representation of the main pathogenetic events in HS is shown in Figure 1.

## 3. (New Medical) Therapeutical Approaches in HS

Several guidelines on HS management have been published by expert groups in Europe, North America, and South America [151]. As recommended by the HS ALLIANCE working group, (a small group of) systemic antibiotics remain the cornerstone for the treatment of patients with moderate-to-severe HS. After conventional treatment failure, the first-line biologic agent in moderate-to-severe HS should be adalimumab, whereas infliximab should be considered as a second-line biologic [152]. IL-36, LCN2, leukotriene A4-hydrolase (LTA4H), MMPs, S100A proteins, G-CSF receptor, and CXC receptors are just some of the possible therapeutic targets for future treatment of HS [153,154]. However, as already mentioned at the beginning, the aim of this article was to describe, on the basis of the most recent scientific evidence, the main current and potential (new medical) therapies for patients with moderate-to-severe HS. The complete list of ongoing clinical trials (recruiting, not yet recruiting, enrolling by invitation, active not recruiting in the clinicaltrials.gov register) focusing on biologic and small-molecule drugs is reported in Table 1.

### 3.1. TNF-α Inhibitors

#### 3.1.1. Adalimumab

Adalimumab (ADA) is a fully human IgG1 monoclonal antibody directed against TNF-α. It is the only treatment for moderate-to-severe HS approved by both the Food and Drug Administration (FDA) and the European Medicines Agency (EMA) [178]. Two multicenter (PIONEER I and II), 36-week, phase 3 trials with two double-blind, placebo-controlled periods were conducted to evaluate ADA efficacy in moderate-to-severe HS patients. The PIONEER I study enrolled 307 patients and the PIONEER II study 326 patients. In the first period (12 weeks), patients were randomized (in a 1:1 ratio) to either ADA (40 mg weekly) or placebo. In the second period (24 weeks), patients were reassigned to receive ADA, 40 mg weekly or every other week, or to placebo. At the end of the first period, at week 12, the Hidradenitis Suppurativa Clinical Response (HiSCR) was achieved by 41.8% vs. 26.0% (PIONEER I, *p* = 0.003) and 58.9% vs. 27.6% (PIONEER II, *p* < 0.001) of HS patients in the ADA and in the placebo groups, respectively. In PIONEER II (this was not observed in PIONEER I), greater significant improvements in lesion count, pain score, and the mean modified Sartorius score were observed in the ADA group than the placebo group. However, PIONEER II allowed patients to continue treatment with antibiotics. ADA was well-tolerated in both studies and adverse events showed no significant differences between the ADA and placebo groups [179]. In a recent systematic review and meta-analysis of five randomized controlled trials (involving 1014 patients), Lu and colleagues evaluated the efficacy and safety of ADA in HS. The authors confirmed that ADA administered weekly showed not only an improvement in clinical response (compared to placebo) but also in symptoms and quality of life of moderate-to-severe HS patients [180]. Concerning long-term ADA efficacy in moderate-to-severe HS patients, a 3-year open-label extension of PIONEER I and II revealed that a sustained response (HiSCR) was maintained in 52.3% of patients treated with ADA, 40 mg per week, through to week 168 [181]. ADA dose intensification (80 mg/week) significantly improved International Hidradenitis Suppurativa Severity Score System (IHS4) score, Pain Index, Hidradenitis Suppurativa Physician’s Global Assessment (HS-PGA), and Cardiff Dermatology Life Quality Index (DLQI) in 14 patients suffering from moderate-to-severe HS with insufficient response or in primary responders with a progressive efficacy loss to 40 mg-weekly ADA. Two patients with HS and concomitant Crohn’s disease (CD) developed psoriatic lesions during this intensified treatment [182]. The safety of ADA administered at 40 mg every other week vs. every week in patients with HS was investigated by Ryan et al., and the researchers reported that adverse event profiles of these two dosing regimens were comparable [183]. Concerning ADA-specific risks in HS, Frew and colleagues, rather recently, in their re-analysis study, have reported that incidence rates of serious infection (2.14 per 100 patient years) and malignancy (0.46 per 100 patient years) in ADA-treated HS patients are comparable to other inflammatory conditions treated with ADA [184]. Recently, Cao et al. have identified CCL16 (human beta chemokine 4 (HCC-4)), calprotectin, and fractalkine as potential predictive biomarkers of adalimumab response in HS patients [185].

#### 3.1.2. Infliximab

Infliximab (IFX) is a chimeric monoclonal IgG1 antibody that recognizes human TNF-α [178]. IFX efficacy in HS was first described in patients undergoing IFX therapy for CD [186,187]. Grant and colleagues assessed the efficacy and safety of IFX therapy in patients with moderate-to-severe HS. In their phase II, randomized, double-blind, placebo-controlled clinical trial, they enrolled a total of 38 patients (26 females and 12 males) randomized to treatment with either IFX (5 mg/kg, *n* = 15) or placebo (*n* = 23) at weeks 0, 2, and 6, followed by a maintenance regimen of IFX every 8 weeks. A decrease of ≥50%, 25%–<50%, and <25% from baseline in HS Severity Index (HSSI) score at week 8 was observed in 26.7%, 60%, and 13.3%, and in 5.5%, 5.6%, and 88.9% of IFX and placebo groups, respectively. IFX was well-tolerated by HS patients who showed improvements in pain intensity and DLQI score with a concomitant reduction in clinical markers of inflammation (ESR and CRP) [188]. A retrospective comparative study of IFX (5 mg/kg at weeks 0, 2, and 6) and adalimumab (40 mg every other week) showed that IFX, in 20 patients with severe, recalcitrant HS, performed better in Sartorius score, DLQI, reduction of ESR and CRP, patient and doctor global assessment, and duration of efficacy [189]. However, IFX optimal dosage remains unclear, and Oskardmay et al. attempted to determine it for patients with HS. The authors, in their retrospective study on 52 patients (73% female), reported that most of the 35 (67%) patients who achieved HS stability were on 10 mg/kg IFX administered every 6–8 weeks [190]. According to these findings, Ghias and colleagues investigated the efficacy of IFX therapy at 7.5 mg/kg every four weeks, with possible dose-escalation to 10 mg/kg (due to inadequate disease control) in moderate-to-severe HS patients. The researchers reported that, at weeks 4 and 12, 20/42 (47.6%) and 17/24 (70.8%), and 6/16 (37.5%) and 6/12 (50.0%) of HS patients achieved a HS-PGA of clear, minimal, or mild (0–2), and at least a 2-grade improvement from baseline, in the IFX 7.5 and IFX 10 cohorts, respectively. No serious adverse events were recorded, and high-dose, high-frequency IFX provided a significant reduction in HS pain [191]. In a recent retrospective multicenter study, the overall drug survival of IFX was 58.3% after 12 and 48.6% after 24 months. Surgery during treatment significantly improved IFX drug survival [192]. A study investigated adjuvant biologic therapy (with either IFX or ustekinumab) after surgical radical resection of HS lesions. Local recurrence was experienced by 19% (4/29) and 38.5% (10/26) of patients in combined and surgery-only groups, respectively [193].

#### 3.1.3. Etanercept

Etanercept is a recombinant human TNF-α receptor p75 Fc fusion protein that blocks the activity of TNF-α [194]. In a prospective open-label phase II study, including 10 HS patients (7 females and 3 males), etanercept 50 mg per week was administered subcutaneously for 12 weeks. At week 12 (compared with baseline), the researchers observed (i) a >50% decrease of disease activity in 6 patients, (ii) a self-evaluation visual analogue scale (VAS) decrease in 7 patients, (iii) a >50% decrease of Sartorius score in 6 patients, and (iv) a decrease of local pain (as early as the first month of etanercept therapy). Disease relapse (8 patients) was reported within 4–8 weeks after the end of etanercept administration [195]. A phase II clinical trial evaluated the safety and efficacy of etanercept 50 mg weekly in moderate-to-severe HS patients. At least a 50% PGA reduction at week 12 (compared to baseline) was achieved in only 3 out of the 15 patients [196]. The only randomized, prospective, double-blind, placebo-controlled trial found no significant efficacy in HS improvement. Twenty patients were treated for twelve weeks with either 50 mg etanercept or placebo twice a week, and after twelve weeks, all patients received etanercept 50 mg twice a week. HS-PGA as clear or mild at week 12 was the primary endpoint. The patient global assessment and DLQI were secondary endpoints. The study revealed, at 12 and 24 weeks, no statistically significant difference in any of these endpoints assessed [197]. In summary, etanercept is not effective for HS treatment [152].

#### 3.1.4. Golimumab

Golimumab is a human monoclonal antibody which binds and inhibits soluble and transmembrane human TNF-α. The use of golimumab has been described in two cases of HS. The first reported a case of a female patient with severe HS and psoriatic arthritis who failed adalimumab, anakinra, and golimumab (50 mg once a month) therapy [198]. The second reported a female patient with concomitant ulcerative colitis, HS, and pyostomatitis vegetans treated with golimumab (200 mg followed by 100 mg every 4 weeks); after 2 months of golimumab therapy, both dermatologic lesions and ulcerative colitis were in remission [199].

#### 3.1.5. Certolizumab Pegol

Certolizumab pegol is a pegylated antigen-binding fragment (Fab) of a recombinant humanized monoclonal antibody targeting TNF-α [200]. In two case reports (involving two male patients), certolizumab pegol appeared to be effective in treating recalcitrant (both patients had previously failed biological therapies) HS [201,202]. A case report of a female patient with concomitant psoriasis, psoriatic arthritis, and HS successfully treated with certolizumab pegol was recently described [203]. In all these case reports, the initial dose was 400 mg, followed by 400 mg every other week (only 1 male patient increased to 200 mg every week) [201,202,203]. In the literature, a successful treatment with certolizumab pegol of a pregnant patient with HS was reported [204].

### 3.2. IL-1 Inhibitors

#### 3.2.1. Anakinra

Anakinra is a recombinant IL-1 receptor antagonist (recombinant IL-1Ra) that blocks receptor activity for both IL-1α and IL-1β. HS patients were administered with a 100 mg subcutaneous QD dose [205]. It has been assessed in an open-label pilot study involving 6 patients (4 females and 2 males) with moderate-to-severe HS who received treatment with anakinra for 8 weeks, with an additional 8 weeks of follow-up therapy. Anakinra, which was well-tolerated, was shown to be effective (in 5 out of 6 patients who completed the 8-week treatment) in significantly reducing both subjective and objective measurements of disease activity [206]. Tzanetakou and colleagues performed the first double-blind, randomized, placebo-controlled clinical trial assessing the safety and efficacy of anakinra in HS. Previous anti-TNF failure was reported by 30% and 44% in the placebo and anakinra groups, respectively. The HiSCR, at week 12, was achieved by 30% (3 out of 10) and 78% (7 out of 9) of patients receiving placebo and anakinra, respectively (*p* = 0.04). At week 24, the HiSCR remained positive in 33% (3 out of 9) and in 10% (1 out of 10) of patients receiving anakinra and placebo, respectively (*p* = 0.28). No serious adverse events were reported. Interestingly, IL-22 production by PBMCs was significantly increased in the anakinra group [207]. André et al. reported long-term efficacy of anakinra therapy in 3 HS patients (2 females and 1 male) [208]. On the other hand, failure of anakinra therapy was demonstrated in a case of severe HS after 3 months of therapy [209].

#### 3.2.2. Canakinumab

Canakinumab is a human monoclonal IgG1/κ anti-IL-1β antibody [210]. In case reports, canakinumab showed mixed results in treating HS. It was administered at different dosage regimens, ranging from 150 mg subcutaneously weekly to 150 mg every 8 weeks [211,212,213,214]. Canakinumab therapy was effective both in improving Sartorius score and reducing pain in 2 patients (1 female and 1 male, both adalimumab-experienced patients) with severe HS without any adverse effect [211]. A very rapid response to canakinumab was observed in a patient with pyoderma gangrenosum and concomitant HS [212]. Contrary to the above-mentioned cases, Sun and colleagues described two cases of two female patients with refractory (they had previously failed anti-TNF-α therapies) pyoderma gangrenosum and HS unresponsive to canakinumab treatment [213]. A case of an obese male unresponsive to canakinumab therapy was reported by Tekin et al. [214].

#### 3.2.3. Bermekimab

Bermekimab (formerly known as MABp1) is a fully humanized monoclonal antibody targeting IL-1α. In a double-blind, 1:1 randomized, placebo-controlled study involving 20 patients (10 in the placebo arm) with moderate-to-severe HS, refractory (primary or secondary failure) to previous anti-TNF treatment or not eligible to receive adalimumab, HiSCR was achieved, at week 12, in 60% and 10% of MABp1 and placebo groups, respectively. After MABp1 treatment, neovascularization and lesion skin depth were both significantly decreased on skin ultrasonography; furthermore, a decrease of circulating IL-8 (and of IL-8 produced from stimulated whole blood) was reported in the MABp1 group. Thus, the researchers suggested that inhibition of neovascularization and modulation of the dysregulated innate immunity might be mechanisms of action of MABp1 [215]. During the open-label extension period, 6 out of 8 patients (75%), originally randomized to placebo treatment, achieved HiSCR after 12 weeks of MABp1 therapy. In both studies, MABp1 was administered as an intravenous infusion of 7.5 mg/kg every 14 days [216]. A phase II open-label study evaluated the safety, tolerability, and efficacy of bermekimab in 42 patients with moderate-to-severe HS. Twenty-four patients (group A) had previously failed at least one anti-TNF therapy, and eighteen patients (group B) were anti-TNF-naïve. In both groups, bermekimab was administered subcutaneously at a dose of 400 mg weekly. After 12 weeks of treatment, HiSCR was achieved by 61% and 63% of group B and group A, respectively. Furthermore, bermekimab was well-tolerated and has been demonstrated to be effective in reducing pain in all HS patients [217].

### 3.3. IL-17 Inhibitors

#### 3.3.1. Secukinumab

Secukinumab is a recombinant fully human monoclonal IgG1/κ antibody that selectively targets IL-17A [218]. In all the following studies, secukinumab was administered at a dosage of 300 mg subcutaneously weekly for the first 4 weeks (days 0, 7, 14, 21, and 28) and every 4 weeks thereafter. In case reports (involving 5 patients, 1 female and 4 males), secukinumab demonstrated efficacy in treating HS, showing a dramatic and rapid (even one week after the first secukinumab injection) improvement in both subjective and objective measurements of disease activity. One male patient had concomitant psoriasis and four out of five patients had previously failed anti-TNF-α therapies. Overall, secukinumab was well-tolerated (1 male patient was diagnosed with oral candidiasis successfully treated with topical miconazole) [218,219,220,221,222]. In an open-label pilot trial involving 9 patients (56% male) with moderate-to-severe HS on secukinumab, HiSCR was achieved, at week 24, in 67% (6/9) of participants. No serious adverse events were reported. In this study, the risk of bias was increased by a relatively high dropout rate [223]. Recently, Reguiaï and colleagues performed a retrospective study on a cohort of 20 HS patients (in whom at least one anti-TNF-α treatment failed or was not tolerated) treated with secukinumab (12 females and 8 males). After 16 weeks, HiSCR was achieved in 75% (15/20) of patients and the average HS-PGA decreased from 4 to 2. Two patients developed CD after 3 and 5 months of secukinumab therapy, respectively. No relapse was observed in the follow-up phase [224]. In the only open-label trial of secukinumab at 2 dosage levels (every 2 weeks or every 4 weeks, administration of 300 mg secukinumab after the loading dose of 5 weekly injections), HiSCR, after 24 weeks, was achieved by 70% of all 20 HS patients (including 6 patients with previous anti-TNF-α exposure). The proportions of patients who achieved a 100% reduction in the sum of abscesses and inflammatory nodules was 15% at week 24. No serious adverse events were detected [225]. However, the effectiveness of secukinumab remains not entirely confirmed. As a matter of fact, a multicentric retrospective study (involving 31 HS patients, 55% female) from 8 Italian centers reported that HiSCR was achieved, at week 28, only by 41% of patients. The researchers recorded an acne-like eruption of the face as the only adverse event [226]. Concerning paradoxical cases of HS, Marasca et al. described a case of secukinumab-induced HS in a male psoriasis patient [227].

#### 3.3.2. Ixekizumab

Ixekizumab is a humanized IgG4 monoclonal antibody that specifically binds IL-17A. Concerning the use of ixekizumab in HS patients, to the best of our knowledge, at the time of writing, only 5 isolated cases have been reported: 2 men with HS and concomitant psoriasis and 3 females (1 with concomitant CD) affected by severe HS. Four out of five HS patients had been previously treated with biologic drugs. Ixekizumab was administered at recommended psoriasis dosage: a subcutaneous injection of 160 mg at week 0, followed by 80 mg at weeks 2, 4, 6, 8, 10, and 12, then 80 mg every 4 weeks. Efficacy and safety of ixekizumab were reported in all cases [228,229,230,231].

#### 3.3.3. Brodalumab

Brodalumab is a recombinant fully human IgG2 monoclonal antibody that specifically targets IL-17RA. Brodalumab was administered subcutaneously at a dosage of 210 mg weekly at weeks 0, 1, and 2, followed by injections at 2-week intervals [232]. Only two case reports have explored brodalumab clinical efficacy and safety in moderate-to-severe HS. The first case was a man successfully treated for his HS after anti-TNF-α failure: after 12 weeks, the number of abscesses and inflammatory nodules decreased from 27 to 9 and IHS4 from 62 to 18. No adverse events were reported [233]. The second was a Japanese man with concomitant psoriasis: brodalumab was well-tolerated and showed efficacy in treating both conditions and improving quality of life [234]. An open-label pilot cohort study was performed to evaluate the safety, tolerability, and efficacy of brodalumab in 10 moderate-to-severe HS patients (50% female). Oral antibiotics, adalimumab, infliximab, secukinumab, and ixekizumab were all previous therapies. The HiSCR was achieved by 100% of patients at week 2 and maintained at both weeks 12 and 24, and 80% of patients achieved the IHS4 category change at week 12. Patients showed significant improvement in vascularity and inflammation (doppler signal), pain, itch, and quality of life. Brodalumab therapy was well-tolerated [232]. However, the researchers observed a cyclical response in HS patients with draining tunnels and they hypothesized that weekly dosing of brodalumab could have provided a better clinical control of HS. Thus, an open-label cohort study was performed to evaluate weekly administration of brodalumab in 10 moderate-to-severe HS patients (all patients had draining tunnels). The HiSCR was achieved by 100% of patients at week 4 (and sustained through to week 24). Compared to bi-weekly dosing, no cyclical response was observed with weekly administration [235].

#### 3.3.4. Bimekizumab

Bimekizumab is a humanized IgG1 monoclonal antibody that selectively neutralizes both IL-17A and IL-17F, functioning as a dual inhibitor [236]. A phase 2 multicenter trial to test the efficacy, safety, and pharmacokinetics of bimekizumab in moderate-to-severe HS patients was completed (ClinicalTrials.gov Identifier: NCT03248531). The results have not yet been published (only presented at EHSF and SHSA meetings), and in preliminary data reported by Zouboulis and colleagues, at week 12, 56.9% of patients in the bimekizumab group achieved the HiSCR compared to 23.7% in the placebo group [153].

#### 3.3.5. CJM112

CJM112 is an anti-IL-17 monoclonal antibody. In a phase 2 clinical trial (ClinicalTrials.gov Identifier: NCT0242117), at week 16, 32.3% of HS patients decreased the basal HS-PGA score by at least 2 points compared with 12.5% of the placebo arm [153,237].

### 3.4. IL-12/-23 Inhibitors

#### Ustekinumab

Ustekinumab is a fully human IgG1/κ monoclonal antibody that binds with high affinity and specificity to the p40 subunit, thus blocking both IL-12 and IL-23. The first 3 cases (2 females and 1 male) of moderate-to-severe HS treated with ustekinumab showed variable results at 6 months [238]. Blok and colleagues studied the efficacy of ustekinumab in 17 moderate-to-severe HS patients (13 females and 4 males). Ustekinumab was administered according to the psoriasis dosing regimen (45 mg administered subcutaneously at weeks 0 and 4, every 12 weeks thereafter; in patients with a body weight greater than 100 kg, a dose of 90 mg may be used). At week 40, the mean of the modified Sartorius scale (mSS) decreased by 46.33%. In a post hoc analysis, HiSCR was achieved by 47% (8 out of 17) of patients at 40 weeks. Interestingly, the researchers hypothesized that low LTA4H concentrations with mild clinical severity might be predictive of ustekinumab efficacy [239]. A multicenter case series involving 10 patients (4 females and 6 males; 2 patients also had CD) with moderate-to-severe HS (9 of whom had received anti-TNF-α therapy, and 1 patient was biologic-naïve) treated with ustekinumab at psoriasis dosage, reported (after a median treatment duration of 48 weeks) a reduction of at least 1 point in HS-PGA and a decrease of ≥2 points in the Numerical Pain Rating Scale (NPRS) in 70% and 80% of patients, respectively. No adverse events were recorded [240]. Romaní et al., in their multicentric (6 hospitals) review involving 14 HS patients (6 of whom had concomitant CD) who had had prior treatment failure with infliximab, adalimumab, certolizumab, or anakinra, retrospectively investigated the efficacy and safety of an intensified dosage of ustekinumab (an initial weight-adjusted intravenous induction dose followed by 90 mg subcutaneously every 8 weeks, according to the ustekinumab regimen approved for CD). At week 16, the HiSCR and a ≥30% decrease of DLQI and VAS of pain were achieved by 50% of patients. DLQI and VAS of pain improved by ≥30% in 71.42% of patients in the absence of significant ustekinumab-related side effects [241]. In accordance with these findings, Sánchez-Martínez and colleagues, evaluating the effectiveness and safety of the ustekinumab dosing schedule approved for CD in 6 adalimumab-experienced HS patients (50% female), found that 50% of patients achieved HiSCR at week 12, without any drug-related side effects [242]. Rather recently, Scholl et al. also reported the potential utility of a high-dose regimen of ustekinumab in their case series including 3 biologic-experienced HS patients (1 female and 2 males) [243]. A case report described a case of HS development during infliximab therapy for CD successfully treated with ustekinumab [244]. In a recent case series, involving 10 moderate-to-severe HS patients treated with 90 mg ustekinumab subcutaneously every 2 months (only 1 patient was administered with 45 mg ustekinumab subcutaneously every 3 months), 90% of patients achieved HiSCR (the average duration of treatment was 17.6 months). Interestingly, according to the authors’ experience, the long-term efficacy of secukinumab may not be influenced by the administration or not of induction [245]. A successful combination of hyperbaric oxygen therapy (6 days per week for 6 weeks) and high-dose ustekinumab (90 mg every 4 weeks) was described in a pediatric obese female HS patient [246].

### 3.5. IL-23 Inhibitors

#### 3.5.1. Guselkumab

Guselkumab is a fully human IgG1λ monoclonal antibody that binds selectively to IL-23. In the literature, case reports and case series reported 19 HS patients (9 females and 10 males) treated with guselkumab at psoriasis dosage (a subcutaneous injection of 100 mg at weeks 0 and 4, then 100 mg every 8 weeks), except for 4 of them treated with 100 mg every 4 weeks. Fifteen patients had previously experienced other biologics: thirteen adalimumab, seven ustekinumab, six secukinumab, four infliximab, and two ixekizumab. Seven patients had concomitant psoriasis (including a paradoxical psoriasiform reaction to adalimumab), and two patients had CD. Guselkumab was well-tolerated, and 14 out of 19 patients experienced improvement or remission after the treatment [247,248,249,250,251,252,253].

#### 3.5.2. Risankizumab

Risankizumab is a humanized IgG1 monoclonal antibody targeting the p19 subunit of IL-23. To the best of our knowledge, at the time of writing, in the literature, only two case reports described the use of risankizumab in a total of three HS patients (two females and one male) after adalimumab failure. The male patient had concomitant psoriasis. Risankizumab was administered subcutaneously, 150 mg at week 0, week 4, and every 12 weeks thereafter. After 3 months of therapy, HiSCR was achieved by both female patients and the male patient presented a complete clinical response of both diseases 16 weeks after starting risankizumab. No serious adverse events were reported (only one mild adverse event occurred: an episode of tonsillitis) [254,255].

#### 3.5.3. Tildrakizumab

Tildrakizumab is a monoclonal antibody that binds to the p19 subunit of IL-23. In a recent case series, involving 5 moderate-to-severe HS patients (40% female, 2 of whom had received prior anti-TNF-α therapy) treated with 100 mg tildrakizumab at weeks 0 and 4 and 200 mg every 4 weeks thereafter, an improvement in abscess and nodule count was demonstrated in all HS patients at week 8 compared to baseline (at week 20, 2 patients reported ongoing improvement). Tildrakizumab therapy was well-tolerated [256]. Furthermore, the researchers reported the efficacy of tildrakizumab in significantly reducing mean abscess and nodule count at month 15 (from baseline) in 9 moderate-to-severe HS patients (4 of whom started with 200 mg every 4 weeks from baseline). One patient experienced a self-limiting episode of diarrhea (probably not related to tildrakizumab treatment) [257].

### 3.6. Complement 5a Inhibitors

#### FX-1

Clinical efficacy of IFX-1, a C5a inhibitor, was demonstrated in a small open-label, single-arm trial in HS patients not eligible for adalimumab therapy: 800 mg of IFX-1 (as a 30 min intravenous infusion) was administered on days 1, 4, 8, 15, 22, 29, 36, 43, and 50. The HiSCR was achieved by 75% of patients at the end of treatment and 83.3% at day 134, and therapy was well-tolerated [258]. However, the subsequent randomized placebo-controlled trial (SHINE) showed, on one hand, a lack of significance in the HiSCR outcome measure between IFX-1 and placebo arms and, on the other hand, a statistically significant efficacy in reducing the number of draining fistulas, supporting the hypothesis that decrease of fistulization might be the major benefit of this treatment [58].

### 3.7. Phosphodiesterase 4 (PDE4) Inhibitors

#### Apremilast

Apremilast is an orally administered small-molecule (a class of medications with low molecular weight) inhibitor of PDE4; the latter, encoded by 4 different genes, is mainly expressed in inflammatory cells (as well as endothelial cells, smooth muscle cells, and keratinocytes), where it degrades cAMP. Apremilast, increasing intracellular levels of cAMP, regulates the inflammatory response, modulating pro- and anti-inflammatory mediators (such as IL-12, TNF-α, and IL-10) [259]. In their randomized controlled trial, including 20 patients with moderate HS (of which 15 were randomly assigned to apremilast therapy at a dose of 30 mg twice daily and 5 to placebo), Vossen and colleagues reported that, at week 16, 53% (8 out of 15) of patients achieved HiSCR in the apremilast group compared to 0% in the placebo group [260]. In a 2-year follow-up of initial responders (8 patients), HiSCR at both the 1-year and the 2-year follow-up visits (compared with baseline) was achieved by all 4 patients who continued apremilast treatment [261]. Weber et al. assessed the efficacy of apremilast (30 mg twice a day) in the management of 9 patients (3 females and 6 males) with moderate-to-severe HS (5 of which had previously failed anti-TNF-α therapies). Three patients did not continue treatment (due to reflux, pre-existing depression, problems with health insurance). Five of the remaining six patients showed a good clinical response, documented by the improvement of mean Sartorius score and the reduction of pain VAS score [262]. Garcovich and colleagues reported 2 male patients affected by severe HS, psoriatic arthritis, and other comorbidities (1 of which was previously treated with biologic agents), successfully treated with apremilast. Patients did not report any adverse events [263]. A case report of a male patient affected by concomitant severe psoriasis and HS, both successfully treated with apremilast (30 mg twice a day), was also described by Lanna et al. [264].

## 4. Conclusions

In conclusion, the current understanding of HS pathogenesis places inflammation as the key actor (the primum movens) in the disease pathogenetic process. Nevertheless, the interplay among genetics, lifestyle, hormonal status, microbiome, and innate and adaptive immune system remains unclear. Furthermore, multicenter and large-scale (racially diverse [265]) OMICs studies are needed to identify novel actors (and possible new therapeutic targets) in HS etiopathogenesis and to improve tailored therapy [266]. In the near future, artificial intelligence (AI) might play a role in HS, firstly in clinical trials, and then becoming useful (for clinicians and patients) in daily clinical practice [267,268].

## Figures and Tables

**Table 1:** The complete list of ongoing clinical trials focusing on biologic and small-molecule drugs.

Intervention	Target	Primary Outcome	NCT Number	Phase	Status
CFZ533 (MA) LYS006 (SM) Placebo	CD40 LTA4H	HiSCR at 16 weeks	NCT03827798 [155]	Phase 2	Recruiting
Risankizumab (MA) Placebo	IL-23	HiSCR at 16 weeks	NCT03926169 [156]	Phase 2	Active, not recruiting
Brodalumab (MA)	IL-17RA	IL-17RA saturation at week 12	NCT04979520 [157]	Early Phase 1	Recruiting
Bimekizumab (MA) Placebo	IL-17A IL-17F	HiSCR at 16 weeks	NCT04242446 [158]	Phase 3	Recruiting
Bimekizumab (MA) Placebo	IL-17A IL-17F	HiSCR at 16 weeks	NCT04242498 [159]	Phase 3	Recruiting
Bimekizumab (MA)	IL-17A IL-17F	Percentage of participants with TEAEs up to week 120	NCT04901195 [160]	Phase 3	Enrolling by invitation
Upadacitinib (SM) Placebo	JAK1/2	HiSCR at 12 weeks	NCT04430855 [161]	Phase 2	Active, not recruiting
INCB054707 (SM) Placebo	JAK1	Mean change in total AN count at 16 weeks	NCT04476043 [162]	Phase 2	Recruiting
Orismilast (SM)	PDE4	Percent change in AN count at 16 weeks	NCT04982432 [163]	Phase 2	Not yet recruiting
Secukinumab (MA)	IL-17A	Time to LOR in HiSCR responders at weeks 52–104	NCT04179175 [164]	Phase 3	Recruiting
Secukinumab (MA) Placebo	IL-17A	HiSCR at 16 weeks	NCT03713619 [165]	Phase 3	Active, not recruiting
Secukinumab (MA) Placebo	IL-17A	HiSCR at 16 weeks	NCT03713632 [166]	Phase 3	Active, not recruiting
Spesolimab (MA) Placebo	IL-36R	Percent change in total AN count at 12 weeks	NCT04762277 [167]	Phase 2	Recruiting
Spesolimab (MA)	IL-36R	Occurrence TEAEs up to week 120	NCT04876391 [168]	Phase 2	Not yet recruiting
Imsidolimab (MA) Placebo solution	IL-36R	Change in AN count at 16 weeks	NCT04856930 [169]	Phase 2	Not yet recruiting
LY3041658 (MA) Placebo	ELR^+^CXC chemokine family	HiSCR at 16 weeks	NCT04493502 [170]	Phase 2	Recruiting
PF-06650833 (SM) PF-06700841 (SM) PF-06826647 (SM) Placebo	IRAK4 JAK1 TYK2 TYK2	HiSCR at 16 weeks	NCT04092452 [171]	Phase 2	Recruiting
KT-474 (SM)	IRAK4	Incidence and severity of TEAEs up to 28 days	NCT04772885 [172]	Phase 1	Recruiting
Guselkumab	IL-23	Changes in levels of cytokines in the skin at week 0 and week 16	NCT04061395 [173]	Phase 2	Not yet recruiting
Avacopan (SM) Placebo	C5aR	HiSCR at 12 weeks	NCT03852472 [174]	Phase 2	Active, not recruiting
Recombinant anti G-CSF receptor (MA)	G-CSF receptor	Incidence of TEAEs (up to 24 weeks) Incidence of AESIs	NCT03972280 [175]	Phase 1	Recruiting
Adalimumab (MA) Monotherapy Adalimumab + surgery	TNF-α	Cost-utility, 2 years	NCT03221621 [176]	Phase 4	Recruiting
Tofacitinib (SM)	JAK1 JAK3	Number of SAEs up to week 18 Change in IFN scores (Baseline and 16 weeks)	NCT04246372 [177]	Phase 2	Recruiting

MA, monoclonal antibody; SM, small molecule; HiSCR, Hidradenitis Suppurativa Clinical Response; IL, interleukin; TNF-α, tumor necrosis factor alpha; LTA4H, leukotriene A4 hydrolase; TEAEs, treatment-emergent adverse events; JAK, Janus kinase; PDE4, phosphodiesterase-4; AN, abscess and inflammatory nodule; LOR, loss of response; IRAK4, interleukin-1 receptor-associated kinase 4; TYK2, tyrosine kinase 2; G-CSF, granulocyte colony-stimulating factor; AESIs, adverse events of special interest; SAEs, serious adverse events; IFN, interferon.
